# Determining the Effect of Rock Strength Parameters on the Breakout Area Utilizing the New Design of the Undercut/Breakout Anchor

**DOI:** 10.3390/ma15030851

**Published:** 2022-01-23

**Authors:** Józef Jonak, Robert Karpiński, Andrzej Wójcik, Michał Siegmund, Marek Kalita

**Affiliations:** 1 Department of Machine Design and Mechatronics, Faculty of Mechanical Engineering, Lublin University of Technology, Nadbystrzycka 36, 20-618 Lublin, Poland; a.wojcik@pollub.pl; 2 KOMAG Institute of Mining Technology, Pszczyńska 37, 44-100 Gliwice, Poland; msiegmund@komag.eu (M.S.); mkalita@komag.eu (M.K.)

**Keywords:** physical and mechanical parameters of rocks, mining extracting tool, FEM analysis, rock breakout failure, rock cone failure

## Abstract

This paper presents the idea and provides an analysis of the rock breakout mechanism utilizing an undercut/breakout anchor. The new design is a modification of a standard undercut anchor, which is commonly found in applications involving steel-to-concrete anchorage. Of particular concern was the effect of the rock breakout strength on the anchor-pullout-induced failure of the rock mass. A numerical analysis was employed to model the effect of the changes to the shape and size of the breakout cones under varying rock strength conditions as a result of modifying the anchor design and loading pattern. The problem in question is pivotal for the potential evaluation of the effectiveness of the said anchor design under the non-standard conditions of its utilization.

## 1. Introduction

The undercutting anchors are primarily considered for use as fasteners in the steel elements of concrete structures [1,2,3]. Anchorage to concrete is a technology executed with the use of a range of bolt types [4,5,6]. The previous work in this area has focused on determining the load capacity of anchors depending on their design, physical and mechanical parameters of the medium in which they are to be installed, and the influence of the installation technology [7,8]. Typical mechanical mining methods cannot always be applied in rock mining. One of the main limitations for the use of mechanical or explosive mining methods in rescue operations is the presence of methane gas in elevated concentrations, which can be explosive [1,9]. Numerical modeling using methods, such as, the finite element method (FEM) [10,11,12,13] or the boundary element method (BEM) [14,15], are very widely used in engineering sciences, especially for the behavior analysis of materials and structures [16,17]. These methods, in combination with experimental studies, enable research towards a detailed understanding of the actual behavior of engineering structures for further optimization [18,19,20,21]. The research to date has been predominantly focused on the efforts to understand concrete failure mechanisms [22,23], developing anchor load capacity estimation methods [24,25], the impact of their design on the pull-out force [26,27], and the impact of the technological parameters of anchorage systems [28,29] on anchor capacity to carry certain loads, including the reach of the breakout area on the free surface of the concrete. The authors of the presented concept of using anchors in mining technology as a method of rock mass separation have conducted many studies aimed at determining the usefulness of the method, estimating the pull-out force, and defining potential areas of its application [30,31,32,33]. Particular attention was paid to the formation of the extent of the failure zone and the potential effect of the interaction of the failure zones (failure cones) on the volume of the loosening rock. Since the volume of the detachment medium depends on the crack propagation that forms and develops under the action of the anchor, the factors affecting the extent of the failure zone were determined. Among these elements, the most important were determined, as follows: the technological parameters of the process (anchor depth, spacing of anchors), the physical and mechanical parameters of rocks, and the geometric parameters of the anchor head. It has been confirmed by field tests that in compact and very resistant rocks (e.g., dolomites), the crack, which accompanies the separation of the so-called failure cone, penetrates (to the free rock surface) along a curve, similar to a parabola. In homogeneous, fine-grained and low strength rocks, there is a pronounced inflection (flattening) in the fracture trajectory, which leads to an increased extent of detachment [34].

Earlier studies have been shown to base the computations on the erroneous traditional 45-degree cone model [8,35], which was, however, replaced due to the said discrepancies between the modeled and the empirical data, by the concrete capacity design (CCD) method for concrete breakout failure [36]. The primary assumption of the latter method [37,38] is that the breakout prism angle is approximately 35 degrees [8]. Characteristically, this failure mode suggests that the minimum distance to the edge enabling the development of the full strength of the quadrilateral pyramid is 1.5 *h*_ef_ (*h*_ef_–effective embedment depth of a headed stud anchor).

By reason of the large discrepancies between the theoretical and the empirical failure modes in relation to the elastic linear and the non-linear fracture mechanics, a number of models have been proposed in order to solve the estimations of the load capacity of anchorage (the pull-out force), and the extent of the breakout area on the free surface (e.g., Eligehausen [22], Piccinin [39], Brincker [7]).

With respect to the assembly techniques, the core interest of the research works and analyses herein was to assess the permissible strength of anchors, based on their design and the conditions intended for their usage [24,25]. These efforts have led to distinguishing two groups of anchor/medium interactions, resulting in the pull-out of the base material (axially loaded anchors expanding at pull out), and leading to the material failure in shear (e.g., anchors placed near the edge of a structure, loaded in the direction tangent to the free surface) [17]. Apart from anchors relying on purely mechanical interlock, it is worth mentioning special design fasteners—chemical anchors. Chemical anchoring provides better performance in applications requiring fastening to distinctly foliated media [6,16]. In recent years, the cone failure of anchor groups (symmetric/asymmetric) has received increased scholarly attention [18].

The finite element method provides vital aid with the analysis of linear and non-linear models of anchoring, nevertheless, in the vast majority of cases, its use is limited to the concrete material (recently treated as a reinforced composite) [19,21,23,40].

This study sets out to broaden the scope of the applications of undercut anchors to include controlled rock destruction and mechanical failure under the modified design of anchors. The primary case for the application of the technique in question in engineering works includes scenarios where classic mining methods are bound to fail or where there are severe limitations involved in the use of standard mining technologies [41].

There are a number of new approaches to modeling the failure of brittle media, especially to analyze the issues of crack propagation in these media [42,43,44]. However, due to the fact that in rock media, the peel-off problems (failure model I) are dominated by cracks with a very defined course (sharply defined), the sufficient accuracy of numerical calculations can be obtained using the elastic-brittle model of the medium. Such a course of failure was observed by the authors at the initial stage of anchor pull-out and the formation of a failure zone in the form of a failure cone. Hence, in the FEM modeling, ABAQUS was mainly used as well as the algorithms suitable for the elastic-brittle fracture analysis of the medium. 

The results from our former studies into rock mass failure under the load of undercut anchors have been presented in scholarly publications [45,46,47]. The key findings include, among other things, the effect of rock strength parameters (Young’s modulus, tensile strength *f*_t_, Poisson number–v) on the breakout prism angle *α* in the initial fracture trajectory [4,34,48,49]. Furthermore, the crack path (including the initial angle of the simplified failure cone) was analyzed depending on the coefficient of friction (Coulomb) of the anchor surface against the rock, the geometry of the anchor head, and the quality issues of anchor assemblages, as well as the wear of an anchor (a potential multiple-use breakout tool) [50,51]. In addition, the influence of the anchor groups and the failure cone interaction in two- [1] and three-anchor systems [45] was numerically investigated. Previous analyses that were conducted by the authors have shown that in the analyzed issue, in the second phase of the development of the cone of destruction, the second fracture mode (shear) of the rock medium appears. Unfortunately, currently the ABAQUS program does not cope well with the correct determination of the crack trajectory in this phase, hence the scope of the analysis was limited to the initial phase of the medium destruction. The problem has been successfully solved [52], however, at the current stage the application of additional, own, complex calculation procedures are required. To date, the aim of the research on undercutting anchors [22,24] has been to determine the pull-out force of the anchor at specified installation conditions (i.e., anchor load) or to determine the strength of the concrete in the resulting engineering structure (pull-out test). In the pull-out test method, the anchor is embedded in a hole made in the concrete and then pulled out using a special device based on the free surface of the concrete structure. The dimension of the support spacing (base ring) is comparable here to the effective anchorage depth (*h*_ef_). This influences the stress field in the zone of formation and development of the so-called failure cone. This makes it difficult to know the actual course of the destruction of the medium structure (including, in particular, the potential extent of the detachment surface on the free surface of the medium), in which the anchor is embedded.

In order to avoid the potential effect of the supports on the formation of the stress field generated by the anchor in the rock medium, the authors, in their previous studies [4,46,47,48], investigated in the field and modeled, using finite element methods, the effect of the undercutting anchor under conditions of minimized influence of supports. The analysis showed that the negligible effect of support interaction occurs for the ratio *R*/*h*_ef_ >3 (*R* = radius of support distribution). An issue of particular interest to the team was the formation of the extent of the failure zone of the medium, which translates into the volume of the potentially detached rock solid. The range of the fracture trajectory depends on the initial propagation angle, which (as shown by studies [34,50,51]) is determined by the geometric parameters of the anchor head, the technology of drilling the anchor hole, and the physical and mechanical parameters of the rock medium.

A disadvantage of the previously used anchor pulling method was the use of very heavy and bulky equipment that was necessary to pull the anchor. This was the reason for changing the pull-out technique from pulling the anchor to spreading the anchor between the bottom of the hole and the near-surface of the undercut hole. This load transfer mechanism leads to stress concentration at the anchor head periphery and simplifies the separation mechanism. The failure process is somewhat similar to that observed during hydraulic fracturing in quasi-brittle shale [39,53].

**Figure 1:**
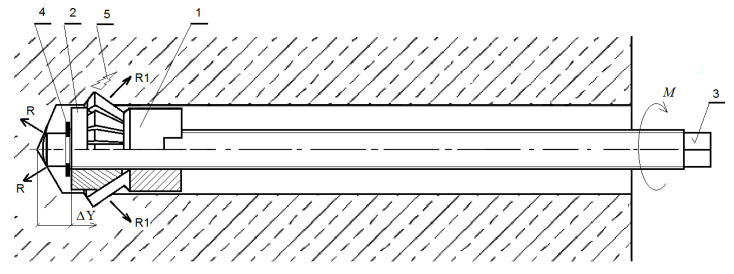
Substrate failure mechanism for the undercut/breakout anchor: 1—undercut nut, 2—expansion teeth, 3—threaded bolt, 4—ring, 5—base material failure propagation, *M*—installation torque, ΔY—displacement of the ring into the sleeve under the installation torque *M*, R—reaction of the threaded bolt against the bottom of the hole, R_1_—cutting teeth expansion into the base material.

Several disadvantages of this solution were identified, such as the need for a large support frame and a hydraulic cylinder to pull out the anchor [1]. In the currently proposed solution, the breakout occurs due to the movement of the undercutting teeth (position 1, Figure 1) over the threaded bolt (position 3, Figure 1) after installation torque M is applied to it [41]. The tip of the bolt (position 3, Figure 1) rests against the bottom of the hole in the rock base material. The displacement Δ*Y* of the undercutting teeth increases, resulting in the advancing deformation of the rock in the contact zone with the expansion teeth (position 2, Figure 1); the gaps (position 5, Figure 1) appear, leading to the breakout of the stone prism. The conical shape of the prism (a quadrilateral pyramid) is a simplification resulting from the basic assumptions of the CCD method [36].

The change in the load transfer from the anchor to the rock medium requires a change in the way this issue is modeled. The question is how this change translates to the potential mechanism of the failure of the rock medium in the proposed technology of rock removal. The doubts that arose were the reason for conducting the analyses in the field, as discussed in this article, and the results of one aspect of the research are presented below.

This study attempts to investigate whether the change in the strength parameters of the base substrate material and the method of loading the undercut anchor affects the initial fracture trajectory and the dimensions of the breakout rock prism. The results will contribute vital information for the effectiveness of the proposed rock breakout technology.

## 2. Materials and Methods

**Figure 2:**
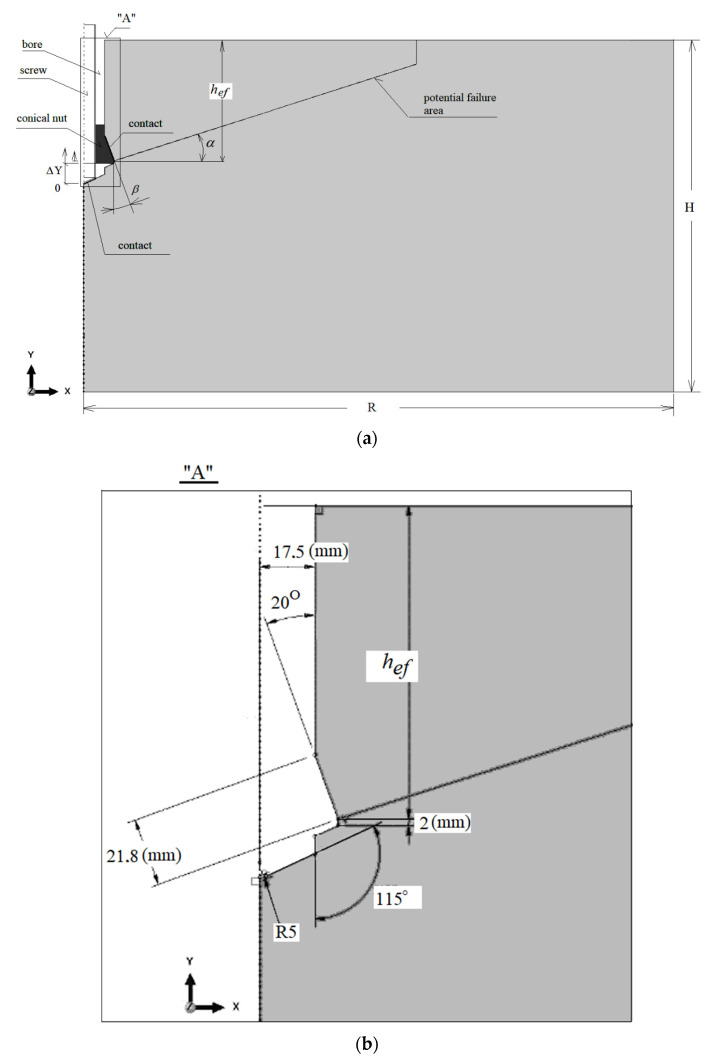
(**a**) The model of the anchor interaction with the base material, *α*—potential base material failure angle, ΔY—controlled (discrete) displacement of the conical nut against the bottom of the hole, *α*— potential breakout prism angle, *β*—angle of the expansion head, *h*_ef_—effective embedment depth, H, R—model dimensions. (**b**) Detail “A”—geometry of the undercut in the base material.

The base material failure analysis was performed using the FEM ABAQUS (Abaqus 2021, Dassault Systemes Simulia Corporation, Velizy Villacoublay, France) software. A flat geometric model was utilized (Figure 2). Due to the axial symmetry conditions, half of the model with the following dimensions was used for calculations: length *R* = 500 mm, height *H* = 300 mm. The effective embedment depth was assumed to be *h*_ef_ = 100 mm. In the model, half the diameter of the hole for the anchor with a dimension of 17.5 mm was mapped (the anchor hole of *φ* = 35 mm corresponds to the dimensions of the undercut in the M20 anchor installation).

Figure 2 (the magnified detail) illustrates the geometry of the undercut in the base rock material required for the installation of the undercut anchor.

The numerical analysis was carried out using the extended finite element method (XFEM) in ABAQUS (Abaqus 2021, Dassault Systemes Simulia Corporation, Velizy Villacoublay, France). One of the main advantages of the method is that it avoids any need for remeshing or geometric crack modelling in numerical simulation, while generating discontinuous fields along a crack and around its tip. The second major advantage of the method is that, by a small increase in number of degrees of freedom, far more accurate solutions can be obtained. The method has recently been extended to nonlinear materials and other disciplines, such as modelling contact and interface, simulation of inclusions and holes, moving and changing phase problems, and even to multiscale analyses [54].

**Interaction Type**: Surface-to-surface contact (standard), discretization method: Surface-to-surface. Finite sliding. Applied minimal Coulomb friction coefficient between the rock and the anchor material (steel) − μ = 0.2.

**Type of material:**

**Sandstone**: Elastic, isotropic. Elastic modulus − E = 14.276 MPa, Poisson’s ratio − *ν* = 0.247, tensile strength − *f*_tI_ = 3.8 MPa; *f*_tII_ = 7.74 MPa; *f*_tIII_ = 17.7 MPa (three theoretical cases were analyzed).

**Steel**: Elastic, isotropic. Elastic modulus − E = 210,000 MPa, Poisson’s Ratio − *ν* = 0.3.

*Damage initiation* in rock material: Maximal principal stress.

*Damage evolution:* Type: energy. Softening: exponential. Damage for traction separation laws: Maximal principal stress damage. Fracture energy = 0.17 N/mm.

**Figure 3:**
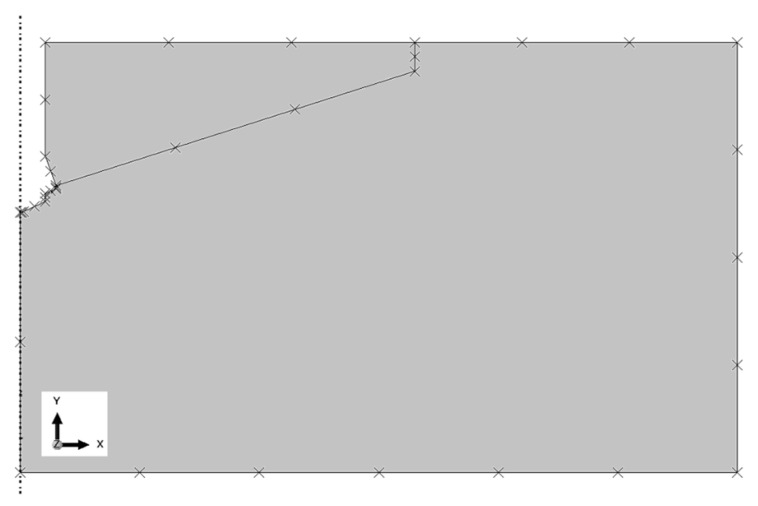
Applied boundary conditions of the base rock material model. Restraints: nodes in the model base U2 = 0, nodes on the left edge.

*Damage stabilization–Cohesive*:

The restraints/boundary conditions are shown in Figure 3. The nodes in the vertical axis of the model under the anchor and at the periphery of the model were deprived of all degrees of freedom (U1 = U2 = U3).

In addition, the following boundary conditions of the model were applied: restraints: nodes in the model base−U2 = 0, nodes on the left edge−U1 = 0 (as shown in Figure 3).

**Figure 4:**
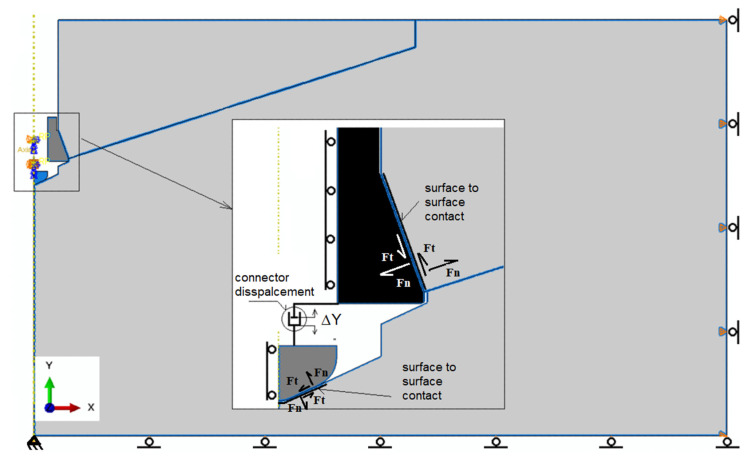
The symmetry conditions of the base material and the system of reference points bound to the anchor along the axis YZ: U1 = UR2 = UR3 = 0, F_n_—normal component, Ft—friction force.

In order to map the kinematic excitation of the anchor head (positions 1 and 2, Figure 1) against the threaded bolt (position 3, Figure 1) that rests on the bottom of the hole, the simulation uses reference points bound to the anchor elements, as in Figure 4. A “connector element” from the ABAQUS finite element library was used to simulate the relative movement of the nut and bolt end as the anchor tears the rock and separates the rock solid, Connection type-AXIAL.

For reference points related kinematically with the anchor elements (U1 = 0, U2 = 0, UR3 = 0), a kinematic excitation in the form of displacement ΔU2_max_ = 10 mm (ΔY–Figure 2) was applied. This allows the head of the anchor to move relative to the tip of the bolt (against the bottom of the hole) only along the OY axis of the adopted coordinate system.

**Figure 5:**
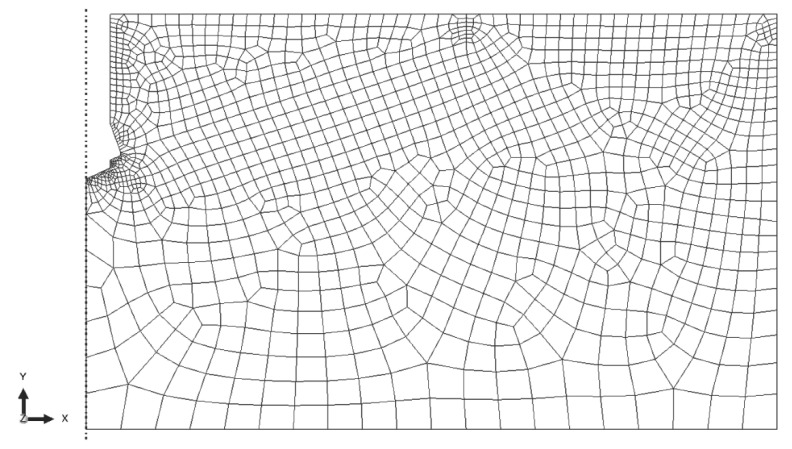
Finite element mesh of the base rock material model.

In the FEM model of the base rock substrate, CAX4R elements were employed—4-node linear elements with reduced integration. The finite element mesh (Figure 5) was constructed with the use of elements with a maximum linear dimension of 25 mm with local densities of up to 1 mm at the edges (Figure 5).

The characteristics of the rock model mesh were as follows: total number of nodes = 1422, total number of CAX4R elements = 1333.

## 3. FEM Model Results

**Figure 6:**
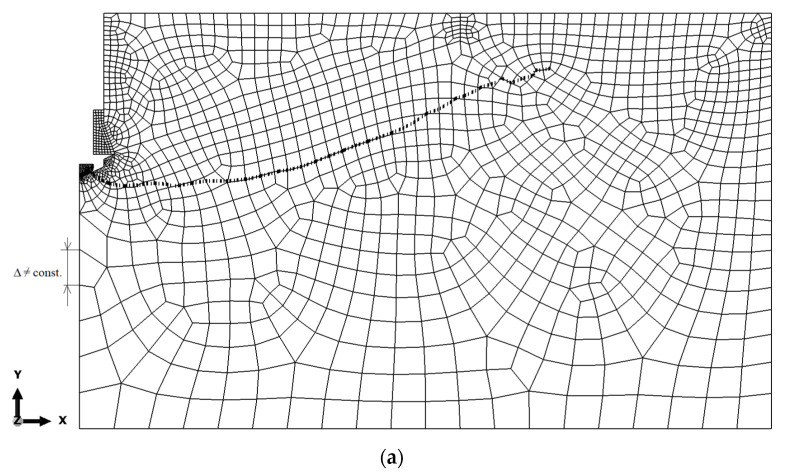

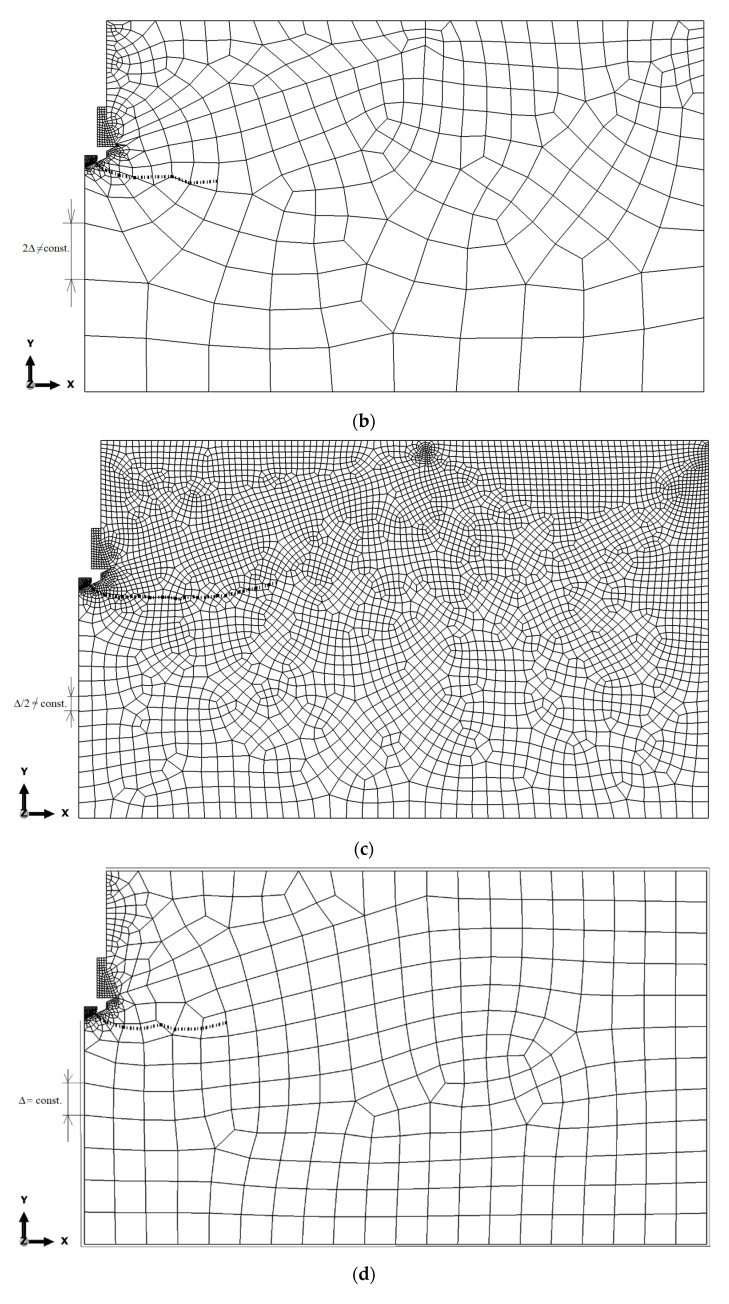

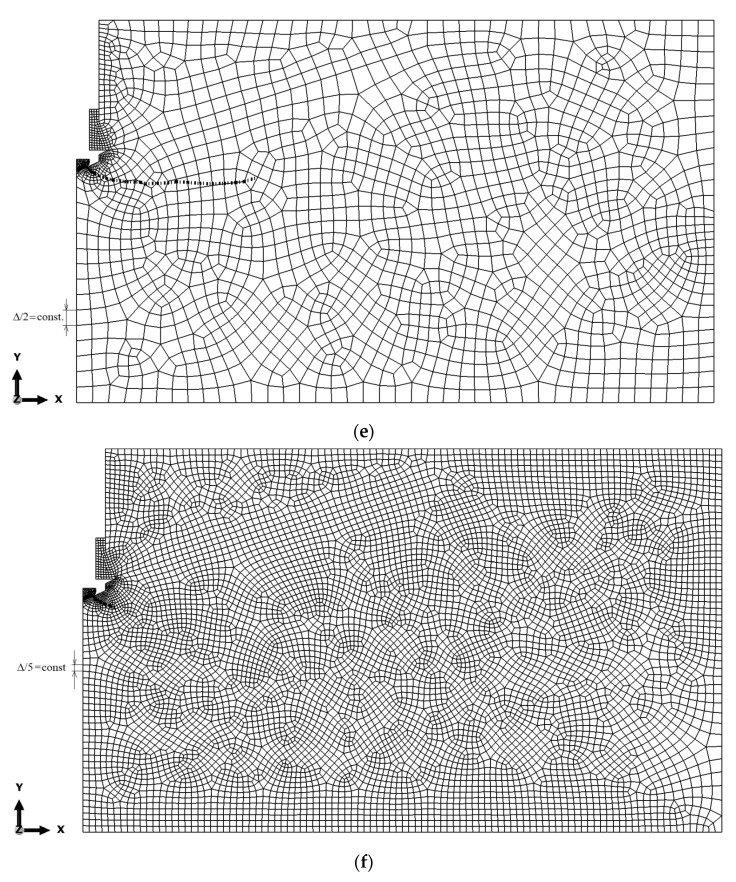
Comparison of the crack path shape for various mesh densities: (**a**–**c**) Δ ≠ const, (**a**) Δmax = 25 mm, (**b**) 2 Δmax = 50 mm, (**c**) Δ/2 max = 12.5 mm; (**d**–**f**) Δ = const: (**d**) Δ = 25 mm, (**e**) Δ = 12.5 mm, (**f**) Δ = 5 mm.

According to one study [55], the size of the FEM mesh is of negligible importance in the X-FEM method. In order to confirm the validity of this thesis for the considered issue, an analysis of the sensitivity of the model to the size of the finite element mesh was carried out (Figure 6). The sensitivity of the model to the finite element mesh discretization method was evaluated using six mesh types. The size of the mesh elements was varied along the edges of the model. In all of the cases of analysis, the dimension of the finite elements in the “contact” zones and the hole edge was constant (1 mm or 5 mm, depending on the contact zone, separated in Figure 4). Variant I (Figure 6a–c) was as follows: the variable length of the mesh elements along the edges of the model (Δ ≠ const.) with thickening near the corners of the model, as shown in Figure 5. Variant II (Figure 6e,f) was as follows: the fixed length of the mesh element side along the model edges (Δ = const). The maximum base dimension of the element side of 25 mm was assumed. The mesh in Figure 5 should be treated as a reference. The subsequent mesh variations were created by increasing/decreasing the dimension of the largest element in the mesh, i.e., when D = 25 mm, resulting in an appropriately condensed or diluted finite element division of the model, as shown in Figure 6. The simulation results are illustrated in Figure 6 (examples of cases).

On the other hand, the tests for a 5 mm mesh were discontinued because the simulation progress was stalled for a long time. The tests for the 25 mm mesh show a large fluctuation in the crack propagation. The conclusion is that the choice of rafting is construction, but the difference between the final and the smallest construction is small. Moreover, the fracture lines in the initial propagation are complementary parts, which can be seen in Figure 6. The fact that the size of the FEM mesh is of negligible importance in the X-FEM method is consistent with the literature.

After evaluating the obtained solutions, it was decided to continue using the mesh according to the model 6a in the analysis, i.e., with the characteristic dimension of 25 mm, taking into account the higher concentration in the area of anticipated failure. 

**Figure 7:**
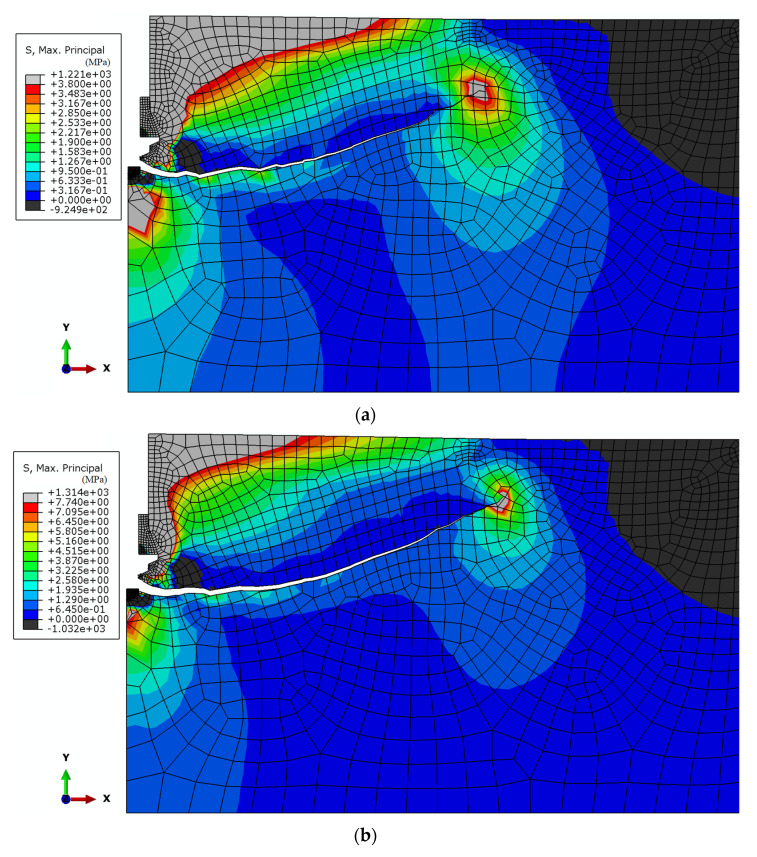

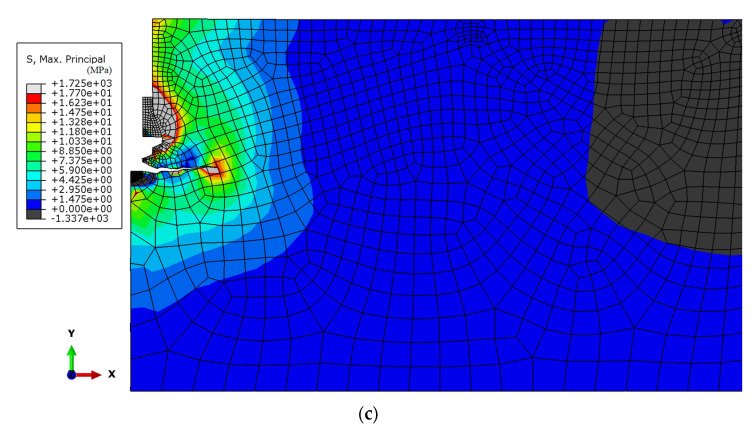
The initial fracture trajectory of the sandstone under the action of the new construction undercutting anchor head, for *h*_ef_ = 100 mm, μ = 0.2. Tensile strength: (**a**) *f*_tI_ = 3.8 MPa; (**b**) *f*_tII_ = 7.74 MPa; (**c**) *f*_tIII_ = 17.7 MPa.

The trajectories of the cracks propagating at the initial stage of the rock material failure were established from the computations (Figure 7).

The impact of the rock breakout resistance (*f*_t_) on the initial fracture trajectory was investigated based on three values of this parameter (as formerly described).

**Figure 8:**
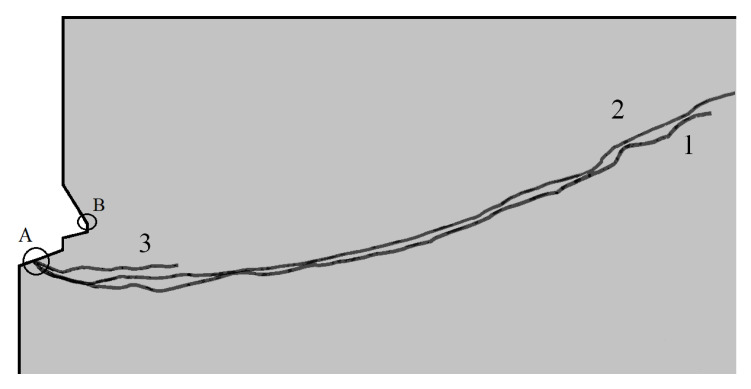
The modeled fracture trajectories: 1—*f*_tI_ = 3.8 MPa; 2—*f*_tII_ = 7.74 MPa; 3—*f*_tIII_ = 17.7 MPa. A—crack initiation point, B—joint initiation point for a typical undercutting anchor.

For the comparative tests, Figure 8 summarizes the obtained fracture trajectories.

Figure 8 illustrates the observable trends. The tendency for deep penetration of the fractures (positions 2 and 3, Figure 8) is negatively correlated with the increase in the rock substrate breakout resistance. Consequently, a significant reduction in the extent of the failure zone on the free surface of the rock substrate is observed, thus decreasing the final volume of the breakout prism (which was also of interest to researchers).

Figure 7 and Figure 8 also show a significant difference in the location of the starting point of the fracture trajectory (Figure 8, point A). This time it is located in the contact zone between the propeller tip and the bottom of the rock hole. In the previous simulations, this point was located at the maximum diameter of the anchor undercut head (maximum undercut diameter in the rock, Figure 8, point B). This point also corresponded to the so-called effective anchor depth (*h*_ef_). The question is whether this is a result of a change in load transfer by the elements of the new anchor design to the rock, resulting in a different model of anchor/rock interaction, or a change in the way the boundary nodes of the model were restrained (which was necessary due to the change in the interaction of the anchor elements with the rock compared to previous models considered in the literature). Further research will be devoted to this issue. 

## 4. Empirical Validation of the Modeling Results

The experimental field tests were conducted in the *Brenna* sandstone mine. These were intended to verify the model data describing the sandstone failure process that was performed with the application of an undercut/breakout anchor adapted from the solutions offered by Hilti (HDA-P M20x200/100).

The strength parameters of the Brenna sandstone are summarized in Table 1.

**Figure 9:**
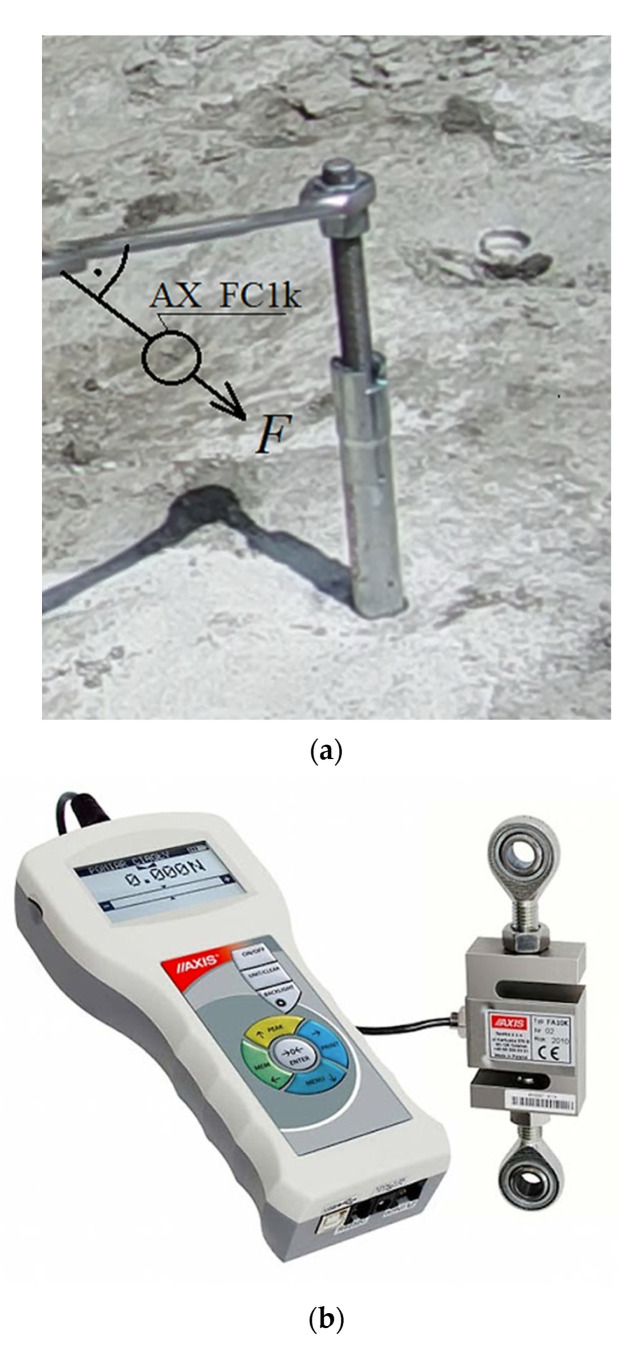
(**a**) The application of the installation torque to the anchor’s threaded bolt (as in position 3, Figure 1) that led to the breakout prism detachment, *F*—force required to turn the threaded bolt in the anchor, (**b**) Electronic force gauge with an external sensor (AX_FC1k).

After the anchor had been installed in the hole drilled in the base rock material, the installation torque was applied to the threaded bolt using a socket wrench (Figure 9). The anchor expanded in the hole and the rock gradually receded under the undercutting teeth (as in Figure 1). In the experiments, the value of the maximum force applied to the anchor bolt was measured using an electronic force gauge with an external sensor (AX_FC1k type, Figure 9b). The sensor was connected to the laptop via the USB port and the changes in the values of the force in question, while the tension was applied, were recorded using the dedicated AXIS FM software [56]. The critical torque was recorded at the moment of the critical opening of the crack, followed by its rapid, uncontrolled development, leading to the breakout prism formation and the torque force value drop to zero. Based on the determined maximum value of the force *F* (Figure 9a) and the length of the wrench with an extension, the value of the maximum torque applied to the anchor was determined. The geometrical relationships in the thread allowed us to determine the maximum axial force in the anchor that led to the pull-out of the failure cone (equivalent to the pull-out force in the case of the former technology). This scope of the analysis, however, will be the subject of a separate future study.

**Figure 10:**
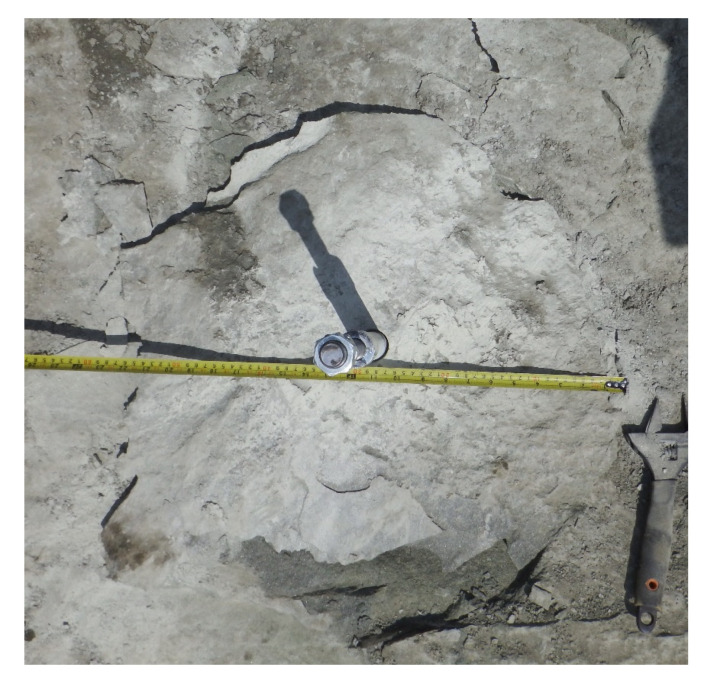
Measurement of the extent of the failure cone on the free surface of the base rock material.

Considering the research objectives of the project, which were to investigate and determine the rock failure mechanism, and to establish the form and the extent of the failure zone in the breakout prism formation using an undercut/breakout anchor, detailed measurements of the extent of the failure on the free surface of the base rock material were correlated with the effective embedment depth (Figure 10).

The trajectory of the fracture propagating in the process of sandstone failure was under scrutiny, as it translates into the volume of the breakout rock prism. The latter is a vital variable for the assessment of the effectiveness of the proposed breakout technology, as well as in terms of the validation of the FEM model results.

**Figure 11:**
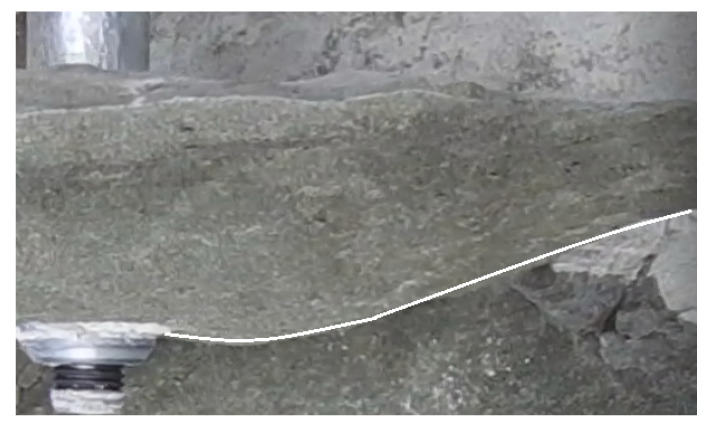
The edge of breakout prism in the sandstone from *Brenna* mine.

It was concluded that the sandstone failure mechanism in the anchor pull-out method [34,47] resembles the failure of the said material at the initial stage of the failure propagating from the bottom of the anchor hole (which is the subject of the present considerations). There is a marked similarity between the crack propagation trajectory that produced the breakout prism here (Figure 11) and the results from the former anchor pull-out study performed in the same sandstone mine (Brenna) [34]. The grey sandstone from the Brenna mine in macro-scale (corresponding to the conditions of the experiment) is characterized by a homogeneous structure, fine grain, and average compressive strength (in contrast to other rock media studied in previous research [34]). In terms of the internal structure and due to its aforementioned homogeneity, the test medium is, in these respects, similar in its behavior to concrete. Hence, this particular rock formation was chosen to perform the experiments because of the high repeatability of the results during the breakout anchor tests. 

Regarding the FEM analysis validation, the fracture propagation shown in Figure 11 (from the field tests) exhibits adequate similarity to the course modeled in the FEM simulation (curve 1, Figure 8). Similarly, the strength parameters of the simulated rock material are, thus, comparable to the mechanical parameters of the sandstone from the *Brenna* mine. Therefore, the correctness of the numerical analysis of the crack propagation in the failure zone induced by the action of the undercut/breakout anchor is further confirmed. The fracture trajectory, shown in Figure 11, has been documented in numerous field and numerical studies involving undercutting anchors [4,34,49]. The above observations show that the mechanism of the destruction of homogeneous rocks, with fine grain size and classified as weaker rock formations, is characterized by a two-stage course of destruction (with an inflection of the trajectory) leading to an increase in the extent of detachment. Moreover, this trajectory, as shown in this study, in its general outline (shape) does not depend on the method of applying the load on the rock (classic undercutting anchor or the anchor in the proposed design and method of applying the load on the rock). The differences in the location of the fracture initiation point require further study, especially numerical, due to the effect of the potential different modeling of the restraints of the propeller nodes and the undercut/breakout head.

## 5. Conclusions

The purpose of the numerical experiment and the relevant field tests was, among other things, to verify the previous knowledge on the mechanism of rock medium failure under the action of undercutting anchors due to the different design of the proposed breakout anchor. The operation of the new anchor is different from the previous undercutting anchors due to a different mechanism of load transfer to the rock mass (breaking of the rock near the bottom of the hole as a result of the expansion of the anchor elements and the lack of support on the free surface), as shown in Figure 1. Supports, in the previous solutions, were necessary to pull out the anchor and could affect the distribution of stresses in the rock, in the zone of anchor action [22,34,49,50,51]. The proposed solution for realizing the destruction of the rock medium structure eliminates these effects.

The proposed structural changes to the anchor and the change in its load transfer mechanism to the rock necessitated the construction of a new mechanical model dedicated to FEM analysis (Figure 4). The question arose as to whether, and possibly how, the response of the system to a different loading regime would change, and what the sensitivity of the model was to the potential changes in the strength of the rock medium (which often occurs during the engineering processes implemented in rock media).

The analysis utilizing the finite element method has given the indication that, also in the case of the undercut/breakout anchor, there is a clearly observed correlation between the sandstone breakout resistance *f*_t_ and the trajectory and dimensions of the failure cone. This further confirms the previous observations from our study of rock failure with the undercut anchor [34]. The growth in the breakout resistance is reflected in the limiting of the failure zone on the free surface of the base material, thereby potentially limiting the volume of the breakout material.

The results from the field tests performed at the *Brenna* sandstone mine site of the trajectory of the anchor-initiated base material failure propagation have been shown to confirm the course determined in the FEM numerical analysis. The two-stage failure propagation, with a notably inflected trajectory, was confirmed for the weak sandstones, as well as the ones found in the *Brenna* mine [34].

Both numerical and field tests have shown that the proposed method of breakout with the use of the undercut/breakout anchor is effective and applicable in field conditions.

The results of the tests confirm the effectiveness of the proposed new stripping method. In addition, the results of both the field and the numerical studies on the mechanism of failure of the medium using of the new design anchor are consistent with the previous results obtained using a classical undercutting anchor. However, the revised method of loading the anchor significantly simplifies the application of load on the anchor to obtain the decoupling effect. This will significantly simplify the area of potential application of this method for the conditions of separation in a limited space (e.g., in the conditions of rescue operations that occur in mine workings after a possible collapse of the workings).

The differences between trajectories one and two may not be large, but it should be noted that the differences between *f*_tI_ and *f*_tII_ strengths are also not very large. Lack of a clear difference in the course of trajectories one and two can also be explained by the relatively low accuracy of the XFEM algorithm in ABAQUS, which was already mentioned in other articles of the authors. However, the location of trajectories one and three clearly show the tendency in the dependence of the damage zone propagation on this parameter of the medium.

The numerical studies, as opposed to the field studies, showed differences in the location of the starting point of the fracture trajectory (Figure 8, point A). It is located, this time, in the contact zone of the propeller tip with the bottom of the hole in the rock. In the previous simulations, this point was located at the maximum diameter of the anchor undercut head (maximum undercut diameter in the rock, Figure 8, point B). This point also corresponded to the so-called effective anchor depth (*h*_ef_). The question is whether this is a result of a change in load transfer by the elements of the new anchor design to the rock, resulting in a different model of anchor/rock interaction, or a change in the way the boundary nodes of the model were restrained (which was necessary due to the change in the interaction of the anchor elements with the rock compared to the previous models considered in the literature). Further research will be devoted to this issue.

## Figures and Tables

**Table 1:** Physical and mechanical parameters of the studied rocks.

Rock	*f*_c_ [MPa]	*f*_t_ [MPa]	*E* [MPa]	*ν* [-]	*K*_IC_ [N/mm^3/2^]	*G*_IC_ [N/m]	Description
“Brenna” sandstone	95.562	3.209	13.727	0.148	25.655	47.946	Sandstone layered, weak

*f*_c_—compressive strength, *f*_t_—tensile strength, *c*—cohesion, *E*—Young’s modulus, *ν*—Poisson’s ratio, *K*_IC_—critical stress intensity factor, *G*_IC_—critical strain energy.

## Data Availability

The data presented in this study are available on request from the corresponding author.
